# Provincial Medical and Surgical Association

**Published:** 1837-07

**Authors:** 


### PROVINCIAL MEDICAL AND SURGICAL ASSOCIATION.

We are happy to observe that this excellent Society continues to grow in extent and estimation. In addition to the Branches mentioned in our last, a flourishing one has been recently established in Somersetshire, under the name of *The Wells, Bridgewater, and Taunton Branch. The Bath District Branch* held its first sectional meeting on Thursday, the 1st of June, at the Bath Institution, under the presidency of Wm. Tudor, Esq. George Norman, Esq. was chosen president for the ensuing year. The number of members of this Branch is at present upwards of sixty.

*The Southern District Branch* held its first annual meeting, at Winchester, on Thursday, the 8th of June, under the presidency of Dr. Crawford. Dr. Fowler, of Salisbury, was chosen president for the ensuing year; and the next meeting was fixed to take place at Salisbury. At this meeting it was resolved to grant a prize of books, value 20*l.*, for the best Medical Essay on a given subject; to be contested for by the members of the Branch, and to be awarded at the annual meeting in 1839. The number of members is at present one hundred.

The anniversary meeting of the Parent Association takes place at Cheltenham, on the 19th and 20th days of July, under the presidency of Dr. Boisragan. Dr. Bardsley of Manchester delivers the Anniversary Address on the 20th, at twelve. The total number of members of the Association now exceeds one thousand.

---

